# High Frequency Jet Ventilation or Mechanical Ventilation for Panendoscopy for Cervicofacial Cancer: A Retrospective Study

**DOI:** 10.3390/jcm12124039

**Published:** 2023-06-14

**Authors:** Stephanie Suria, Raphaëlle Galy, Lauriane Bordenave, Cyrus Motamed, Jean-Louis Bourgain, Joanne Guerlain, Antoine Moya-Plana, Jamie Elmawieh

**Affiliations:** 1 Department of Anesthesiology, Gustave Roussy, Paris-Saclay, F-94805 Villejuif, France; stephanie.suria@gustaveroussy.fr (S.S.); raphaelle.galy@gmail.com (R.G.); lauriane.bordenave@gustaveroussy.fr (L.B.); bourgainjl@orange.fr (J.-L.B.); jamie.elmawieh@gustaveroussy.fr (J.E.); 2 Department of Cervico Facial Oncology, Gustave Roussy, Paris-Saclay, F-94805 Villejuif, France; joanne.guerlain@gustaveroussy.fr (J.G.); antoine.moya-plana@gustaveroussy.fr (A.M.-P.)

**Keywords:** panendoscopy, high frequency jet ventilation, airway management, cervicofacial cancer

## Abstract

Introduction—the upper airway panendoscopy, performed under general anesthesia, is mandatory for the diagnosis of cervicofacial cancer. It is a challenging procedure because the anesthesiologist and the surgeon have to share the airway space together. There is no consensus about the ventilation strategy to adopt. Transtracheal high frequency jet ventilation (HFJV) is the traditional method in our institution. However, the COVID-19 pandemic forced us to change our practices because HFJV is a high risk for viral dissemination. Tracheal intubation and mechanical ventilation were recommended for all patients. Our retrospective study compares the two ventilation strategies for panendoscopy: high frequency jet ventilation (HFJV) and mechanical ventilation with orotracheal intubation (MVOI). Methods—we reviewed all panendoscopies performed before the pandemic in January and February 2020 (HFJV) and during the pandemic in April and May 2020 (MVOI). Minor patients, patients with a tracheotomy before or after, were excluded. We performed a multivariate analysis adjusted on unbalanced parameters between the two groups to compare the risk of desaturation. Results—we included 182 patients: 81 patients in the HFJV group and 80 in the MVOI group. After adjustments based on BMI, tumor localization, history of cervicofacial cancer surgery, and use of muscle relaxants, the patients from the HFJV group showed significantly less desaturation than the intubation group (9.9% vs. 17.5%, OR_a_ = 0.18, *p* = 0.047). Conclusion—HFJV limited the incidence of desaturation during upper airway panendoscopies in comparison to oral intubation.

## 1. Introduction

Cervicofacial cancers are among the most frequent cancers worldwide [1]. Diagnosis of this type of cancer requires a complete nasopharyngolaryngoscopy and esophagoscopy under general anesthesia with biopsies of any detected lesions since synchronous cancer is frequent [2,3]. The main anesthetic challenge is the management of the airway as there is a high risk of a difficult airway management (cancer, previous oral or cervical cancer surgery or cervical radiotherapy [4,5]). Different airway strategies have been described [2,6] but we could not find any French or international recommendations about optimal airway management during a panendoscopy for cervicofacial cancer patients. First, mechanical ventilation with orotracheal intubation (open system) is often used [7]. This technique allows complete protection of the airway and guaranties optimal oxygenation after intubation, but the tube requires a direct laryngoscopy and could decrease the quality of the first surgical assessment because of a possible risk of trauma and bleeding. In addition to these risks during the procedure, transient hypoxemia may occur because of the increased incidence in difficult ventilation and intubation in these patients [5].

Secondly, panendoscopy can be performed with apnea and intermittent mask ventilation (open system) [8]. The technique requires regular interruption for ventilation during the procedure [9]. The duration of the apnea can be prolonged with transtracheal oxygen [10,11] or high-flow nasal oxygen therapy (Optiflow™, Fisher & Paykel Healthcare, Auckland, New Zealand) [12]. High-flow nasal oxygen therapy allows a mean duration of apnea of 20 minutes, which could be sufficient to terminate the panendoscopy. However, this method may not allow for the complete elimination of carbon dioxide and could yield hypercapnia at the end of the procedure. In the same context, Habrial et al. described a French survey based on a spontaneous breathing technique for this procedure [13]. This technique exposed a risk of failure (23%) in obese patients, patients with a history of difficult intubation and laryngeal tumor patients, which is probably not adequate for our population of patients [13]. In addition, some patients in this last survey might have been intubated and had spontaneous breathing which further complicated the conclusions.

Third, high frequency jet ventilation (HFJV) with a transglottic or transtracheal catheter can be used for oxygenation during the panendoscopy [14,15]. This mode of ventilation provides adequate oxygenation and carbon dioxide elimination during general anesthesia. The jet ventilator generates a high-pressure gas (>1 Bar) by a small diameter injector at a frequency of 120/min. Passive expiration is maintained by the upper airway, which should remain clear. The primary risk is barotrauma (pneumothorax, pneumomediastinum and subcutaneous emphysema) mainly due to the obstruction of the upper airway during expiration [16]. Prevention of barotrauma requires the monitoring of the end-expiration tracheal pressure and the control of the insufflation according to this pressure [17]. The transglottic or transtracheal catheter is connected to the jet ventilator and both allow optimal conditions for surgery, although the transtracheal catheter does not require a direct laryngoscopy [18].

At the Gustave Roussy Institute, we have for many years routinely used the JVHF with a transtracheal catheter inserted into the intercricothyroid space. We usually perform about 800 panendoscopies with HFJV per year. During the spring of 2020, the COVID-19 pandemic imposed important modifications to the management of the airway. The risk of contamination of the staff members during maneuvers of ventilation and intubation was very high. As passive expiration air with JVHF flows directly into the operating room air without a filter and the risk of dissemination and aerosolization of viruses was very high [19,20]. International guidelines about airway management were published [21] and, subsequently, HFJV was contraindicated. According to these recommendations, we decided to realize tracheal intubations for all the surgeries and procedures requiring mechanical ventilation from April 2020. Panendoscopy procedures, which were essential for the diagnosis of cervicofacial cancers, were maintained during the COVID-19 pandemic. 

The primary objective of this retrospective study was to compare JVHF with the transtracheal catheter and mechanical ventilation with orotracheal intubation for panendoscopy. The primary endpoint was the occurrence of desaturation (SpO_2_) less than 90% for more than one minute during HFJV or mechanical ventilation. The quality of the surgical examination was the secondary endpoint. We also analyzed the incidence of severe desaturation (SpO_2_ < 80%). 

## 2. Materials and Methods

### 2.1. Study Design

After approval by our institutional review board on 10 December 2020, we conducted a retrospective study in our teaching cancer hospital between 1 January and 31 May 2020, with the exclusion of March 2020 because of the pandemic situation in France in which all non-emergency procedures including diagnostic panendoscopies were postponed in most hospitals and resources were dedicated to the pandemic situation. 

We included all patients scheduled for a routine panendoscopy during the defined period. In our hospital, airway management for this procedure was based on HFJV before the COVID-19 pandemic (Group 1: HFJV, 1 January to 29 February 2020) and Mechanical Ventilation with Orotracheal Intubation during the COVID-19 pandemic (Group 2: MVOI, 1 April to 31 May 2020). 

Exclusion criteria were patients under 18, those having tracheostomy before or immediately after the procedure; and those having a crossover switch (oral intubation or jet ventilation) performed for any reason.

### 2.2. Anesthetic Management

All panendoscopy patients were under the direct supervision of an experienced staff anesthesiologist and a senior cervicofacial surgeon who were always present in the operating room. After insertion of an intravenous line, patients were monitored during the procedure with electrocardiography, non-invasive blood pressure, oxygen saturation, and capnography (in case of tracheal intubation) and objective neuromuscular monitoring was performed using a continuous objective train-of-four accelerometric monitor (values of this monitor and other vital signs were directly stored in a one minute repetition time in the Centricity Anesthesia GE (CAGE) database). The anesthesia electronic file consists of one condensed print digital format (PDF) saved in the electronic medical file of the patient, and another file containing maximum details, especially all automated recordings of every minute data stored on the CAGE server. This database can deliver all data with every minute detail upon request in an Excel sheet for one patient or deliver a list of files upon a specified sequential query language (SQL) request; JLB has specific and adequate training to administer this database and formulate specific requests.

Group 1: HFJV (before COVID-19 pandemic)

After preoxygenation for an end-tidal fraction of oxygen (Et02) > 90%, general anesthesia was induced and maintained with propofol and remifentanil (target-controlled infusion (TCI) system). A transtracheal cannula was inserted in the intercricothyroid space respecting a strict sterile method under the direct supervision of the senior anesthesiologist using a Seldinger technique with a 16 g needle and a plastic catheter. Lidocaine 2% 4ml as local anesthesia was injected into the trachea to decrease the cough reflex. The catheter was connected to the jet ventilator (Monsoon™, Acutronic, Bubikon, Switzerland), secured with a transparent fixation, and the jet ventilation was started with pre-adjusted parameters (Peak Inspiratory Pressure: 13 cm H_2_O, Work Pressure 1.5 bar, Pause Pressure 6 cm H_2_O, Inspiratory Time 30%, Frequency 110/min, Inspired Fraction of Oxygen 100%) [15,22]. During the procedure, both the anesthesiologist and surgeon focused, as much as possible, on securing expiration as the jet ventilator would stop ventilating in case of obstruction. The intercricothyroidian catheter was removed when the patient was awake with a clear airway and adequate spontaneous respiration. 

Group 2: MVOI (during COVID-19 pandemic)

Specific personal protective equipment was used in the operative room and in the hospitalization unit: head protection with a hood cap, FFP2 (N95) mask, safety glasses, gown, and gloves. All patients had preoperative SARS-CoV-2 negative testing with nasopharyngeal reverse-transcription polymerase chain reaction (RT-PCR). The panendoscopy was performed under general anesthesia with tracheal intubation. After preoxygenation for Et02 > 90%, general anesthesia was induced by propofol and remifentanil (TCI system). Muscle relaxation was recommended with short-action drugs due to the COVID-19 pandemic [21]. Mask ventilation was not recommended during induction because of the risk of dissemination and most of the time a rapid sequence induction was performed. After successful orotracheal intubation with videolaryngoscope or laryngoscope, the tube was connected to the ventilator (Dräger Perseus^®^, Lübeck, Germany). In the case of trismus, an awake fiberoptic nasotracheal intubation (aScope™, Ambu, Ballerup, Denmark) was performed under remifentanil, TCI. General anesthesia was maintained with propofol or the volatile anesthetic Sevoflurane. After the procedure patients were extubated in the operating room with the presence of a senior anesthesiologist and sometimes the cervicofacial surgeon if a difficult extubation was anticipated. Standard extubation criteria were used, such as adequate neuromuscular recovery (train-of-four ratio higher than 90%), respiratory efficiency with adequate respiratory frequency, tidal volume, and smooth cooperation. 

### 2.3. Data Collection

The anesthesia files (PDF) are integrated into the patient’s electronic medical file at our hospital. In addition, detailed recordings of all data were extracted from our Anesthesia Information Management System (AIMS) and the CAGE database, which permitted a data check every minute [23,24]. The clinical data were collected from the computerized medical report. Demographic data (age, gender, height, and weight), preoperative morbidities, prediction of difficult intubation, ventilation criteria, cancer localization, and any history of cervical radiotherapy or surgery were identified in the preoperative anesthetic records. Intraoperative records were analyzed to identify the conditions of facial mask ventilation and intubation (easy, difficult, and impossible) and the mode of ventilation (HFJV or mechanical ventilation after intubation). The doses of the administered medications and the duration of the surgery were recorded manually. Perioperative incidents were analyzed. 

Desaturation was arbitrarily defined by the intraoperative decrease in the SpO_2_ to less than 90% for more than one minute. Analysis and extraction of the computerized anesthetics reports from the CAGE database allowed the collection of SpO_2_ every minute, these values were analyzed individually in combination with other clinically relevant anesthesia file records to eliminate artifacts, for example, a single value without prior stepwise decrease was not considered relevant. In addition, our anesthesia recording system program permits manually adding each complication and its side effects including desaturations in the computerized file records of each patient. A SpO_2_ less than 80% was defined as deep desaturation using the same criteria combined with clinical situations eliminating artifacts and false values allowing determination of the cause of the incident. 

The surgeon defined the quality of the surgical examination: optimal or non-optimal, which was extracted from the surgical report.

### 2.4. Statistical Analysis

No statistical power calculations were performed prior to the study since the sample size in this study was based on the use of data from an available sample.

Patient characteristics were described using the number and proportion of qualitative variables and mean with standard deviation for continuous variables. Standardized differences were used to compare the distribution of baseline variables between the two groups. A standardized difference of less than 0.10 suggests balanced baseline characteristics between patients [25].

A Chi^2^ test was used to compare the events in each group. A multivariate analysis, adjusted for unbalanced demographic and intraoperative parameters in the two most relevant groups, was performed by logistic regression. The quality and duration of the examination were analyzed using a Fisher test.

The results given for the continuous values are in terms of mean (standard deviation) and in terms of number (percentage) for the discrete values. The adjusted odds ratios (ORa) are expressed with their 95% confidence interval (95% CI). The results are considered significant if *p* < 0.05. Missing values were extrapolated using the multiple imputation method. The statistics were performed using R software.

## 3. Results

**Figure 1:**
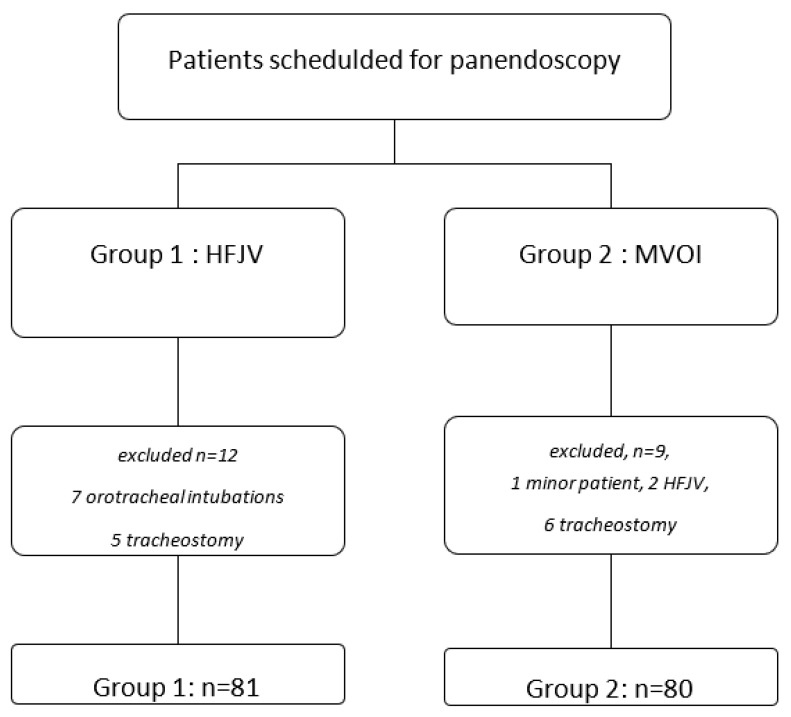
Flow chart. HFJV: High Frequency Jet Ventilation, MVOI: Mechanical Ventilation with Orotracheal Intubation Group 1: 1 January 1 to 29 February 2020, Group 2: 1 April to 31 May 2020.

One hundred and eighty-two patients were identified. The flow chart is presented in Figure 1. After the exclusion of 12 patients in Group 1 and 9 in Group 2, 161 patients were analyzed. All remaining patients had their panendoscopy as planned and no procedure was canceled. The mean missing data for the overall parameters extracted from our AIMS system was 6% for oxygen saturation; we had an overall mean missing data of 5%, therefore, no additional patient files were excluded.

Posthoc power analysis was performed using R software with the epi R package; the incidence of desaturation in group HFJV was 0.099 and the percentage of desaturations in group MVOI was 0.17 with a sample size of 81 and 80, respectively. With an alpha risk of 0.05, the power was calculated as being 80%.

The characteristics of the patients are presented in Table 1. The groups are balanced and comparable except for the body mass index (BMI), the history of cervicofacial cancer surgery and the tumor localization. The mean duration of the procedure was not different between the groups (Group 1: 56.9 min, Group 2: 59.5 min, *p* = 0.53). The administered anesthesia drugs are reported in Table 2. In Group 2, the depth of anesthesia was maintained with 2% inhaled Sevoflurane for 42 patients (52.5%). Administration of a neuromuscular blocker was not systematic in Group 2 and a significant difference was observed in the incidence of administration of the neuromuscular blockers between the groups; we included this factor in the adjusted multivariate analysis.

**Figure 2:**
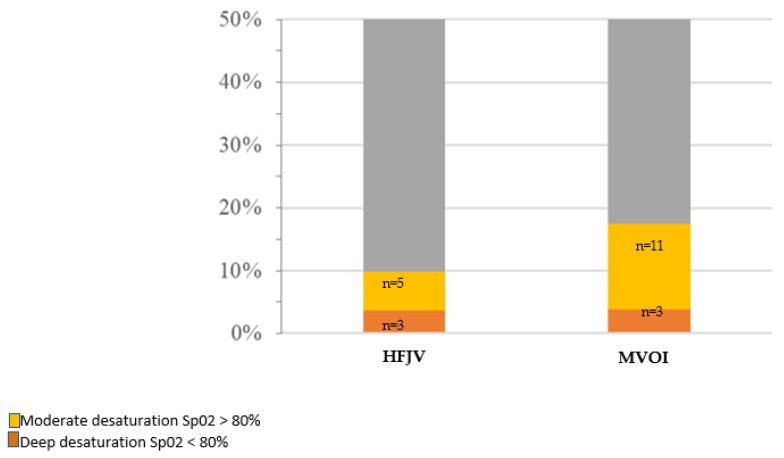
Desaturation according to the Ventilation: HFJV or MVOI. HFJV: high frequency jet ventilation, MVOI: mechanical ventilation with orotracheal intubation.

Twenty-two desaturations were observed: 8 (9.9%) in group HFJV and 14 (17.5%) in group MVOI (Figure 2). In the group HFJV, three deep desaturations (SpO_2_ < 80%) were observed: one kinking of the jet ventilation catheter responsible for a long apnea, one complete obstruction of the airway with failure of the jet ventilation requiring an emergency tracheostomy, and one orotracheal intubation performed by the surgeon due to partial efficacy of the HFJV. We did not report any barotrauma or laryngospasm. In group MVOI, we observed three deep desaturations (SpO_2_ < 80%). Among them were two laryngospasms during a difficult intubation. The third event was due to a difficult intubation without laryngospasm. None of the desaturations were associated with arterial hypotension. 

**Figure 3:**
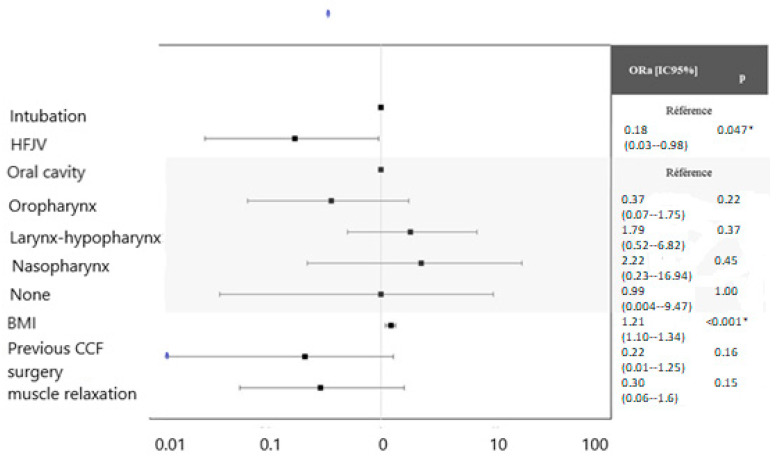
Multivariate Analysis: adjusted odds ratio and confidence interval. * SMD > 0.1: Adjusted variables for the multivariate Analysis.

Figure 3 displays the adjusted odds ratio (ORa) on the confounding factors previously identified (BMI, previous history of cervicofacial cancer surgery, cancer localization, and use of muscle relaxants). After adjustment, the proportion of desaturation was significantly lower in the HFJV group (ORa = 0.18, *p* = 0.047). The BMI was an independent risk factor of desaturation during the panendoscopy (ORa = 1.21, *p* < 0.001). The cancer localization, the history of cervicofacial surgery, and the use of muscle relaxants were not significantly associated with the desaturation after adjustment.

We did not observe any difference between the groups regarding the quality of the surgical examination, as the number of non-optimal conditions was one (1.2%) in group HFJV and six (7.5%) in group MVOI with 95% OR 0.16 [0.0033–1.3292], *p* = 0.06.

## 4. Discussion

In this retrospective study, we observed significantly fewer desaturations with HFJV than the mechanical ventilation with orotracheal intubation during panendoscopy for cancer. We reported a high proportion of desaturation during this procedure for cervicofacial cancer patients. This adverse event is common during this very specific surgery, which is characterized by the management of a shared airway space by surgeons for high-risk patients.

We observed eight (9.9%) desaturations in the group HVJF. We usually performed HFJV for this surgery. Cozine and Ross-Anderson have reported most complications due to barotrauma [26,27]. We did not realize a systematic chest X-ray to detect pneumothorax or pneumomediastinum, as this was an assessment of routine practice. In the group HFJV, most of the desaturations occurred during the “safe stop” of the jet ventilator because of the high tele expiratory pressure. This alarm is due to an insufficient expiration of the gas because of an airway obstruction. It is essential to control the permanent expiration of the patient during jet ventilation to limit the risk of apnea and desaturation. In group MVOI, desaturation occurred in 17% of the patients. The specific conditions of the intubation could explain this relatively high percentage of desaturation: rapid sequence intubation in high risk of difficult intubation and ventilation patients with a history of cancer or cervical radiotherapy and surgery [28,29]. It should be noted that due to the the COVID-19 pandemic, the French Society of Anesthesiologists recommended a rapid sequence intubation without ventilation, and some desaturations could have been the direct result of this type of induction in which some patients with low oxygen reserve, difficult ventilation, or intubation could have a desaturation; we do not believe that these desaturations were related to the experience of the anesthesia providers, as a senior anesthesiologist was always present at the induction for panendoscopies.

No files were deleted as all patients had their panendoscopies; the percentage of missing data (5%) with regard to our primary objective (desaturations) was comparable to our previous investigations [23,24].

Twenty-five patients had ventilation with a facial mask despite the rapid sequence intubation; among them, ventilation was difficult for three and impossible for two patients. These patients presented deeper desaturations.

Only 82% of the patients received muscle relaxants while guidelines recommended the systematic use of muscle relaxants during the COVID-19 pandemic [21,30]. Among paralyzed patients, the dosage of muscle relaxants administered did not appear to be adequate. Supply difficulties with muscle relaxants may explain this discrepancy. Only 10 patients were intubated with video laryngoscopy while guidelines recommended systematic video laryngoscopy during the COVID-19 pandemic [21,30]. In our institute, batteries for the video laryngoscope were produced in China and were not available at the beginning of the pandemic.

In group MVOI, two deep desaturations were caused by a laryngospasm. This kind of complication is frequent during an apnea panendoscopy [9] and is correlated with an inadequate depth of anesthesia which is not always monitored during the procedure. Doses of hypnotic agents cannot be compared between the groups because the anesthesia protocol was not standardized: intubated patients received Propofol alone or in association with Sevoflurane, although patients with jet ventilation received only Propofol.

One of the patients in group MVOI presented severe laryngospasm. A transtracheal jet ventilation catheter was inserted and helped to restore oxygenation. Jet ventilation is effective in emergencies but requires training [4,31,32].

The multivariate analysis was performed by adjusting parameters according to the following: BMI, the antecedent of ENT surgery, localization of the lesion, and use of muscle relaxants. These parameters were significantly different in the two groups. The dosage of intraoperative Propofol and Remifentanil was also unbalanced. We did not adjust these parameters because some of the intubated patients received Sevoflurane, which did not allow for comparing the hypnotic dose. The dosage of Remifentanil did not seem relevant, and the statistical methodology of the study did not allow us to adjust more than four factors.

The multivariate analysis highlighted BMI as an independent risk factor for desaturation. In addition, being overweight or obese was common in patients with the deepest desaturations. Indeed, this risk was already well established by numerous studies and was related to a restrictive syndrome and the occurrence of atelectasis in obese people [33,34]. It can be prevented by proclive preoxygenation [35,36] in positive pressure [37,38,39,40] and the application of an intraoperative PEEP [41]. Bourgain et al. showed that transient hypoxemia in obese patients was also more common under jet ventilation without barotrauma [16,42,43]. We admit that we had a high percentage of desaturation in the MVO2 group; we do not believe that this incidence was related to the experience of the anesthesia providers, as a senior anesthesiologist was always present during the procedure in our specialized high-turnover cancer hospitals. We assume that this relatively high incidence has multiple causes, including comorbidities, the presence of laryngeal tumor, and the fact that because of the COVID-19 pandemic anesthesia protocol recommendations that all orotracheal intubations were performed under a rapid sequence regimen without ventilation yielding an increased risk of desaturations. On the other hand, the fact that only 82% of patients had muscle relaxants, despite recommendations, could also partially explain this excessive incidence of desaturation; indeed, successive French guidelines recommend that muscle relaxants should be administered for tracheal intubation [30].

The quality of the examination was not significantly better in the jet ventilation group in our study; this result was probably related to a lack of power of the present the study for this objective. Some other shortcomings included a lack of consideration of other important clinical outcomes, such as length of stay and possibly late complications including cardiac complications. It should be noted that this procedure is performed nowadays in an ambulatory base in our hospital and an outcome such as length of stay in this context is no more a key parameter for such an assessment, as neither is the length of stay in the postanesthetic care unit.

Finally, this retrospective and single-center nature study did not allow for high reproducibility. Transtracheal jet ventilation is widely practiced at the Gustave Roussy Institute: we perform nearly 800 panendoscopies under JVHF per year. This expertise allows us to observe a low level of complications such as mild subcutaneous emphysema without serious complications in recent years (no pneumothorax reported since 2014). Indeed, the rate of complications was directly inversely related to the number of procedures performed under jet ventilation each year [26]. The COVID-19 pandemic was an opportunity to challenge our practices. However, in light of our study, HFJV was reintroduced as soon as possible for panendoscopies. It is an efficient and secure technique to manage the airways of these patients during this procedure. The applicability of this technique in centers with low turnover could be questioned as this technique needs expertise in order to be performed routinely.

## 5. Conclusions

In this study, transtracheal jet ventilation was associated with less desaturation during upper airway panendoscopies. This ventilation method was safe and suitable for this procedure; future randomized studies should also consider other methods and clinical parameters.

## Figures and Tables

**Table 1:** Demographic characteristics of the population.

	Group 1 (HFJV) *n* = 81	Group 2 (MVOI) *n* = 80	SMD
Age years, mean (± SD)	61.7 (±13.8)	62.1 (±12.8)	0.031
Men *n (%)*	54 (66.6)	54 (67.5)	0.018
BMI Mean, (± SD)	24.8 (±5.19)	25.4 (±5.68)	0.116 *
ASA Score *n* (%)			0.060
1	9 (11.1)	10 (12.5)	
2	51 (62.9)	51 (63.8)	
3	21 (25.9)	19 (23.8)	
Mallampati Score *n* (%)			0.065
1–2	62 (76.5)	59 (73.8)	
3–4	19 (23.5)	21 (26.2)	
Mouth opening < 3 cm or thyromental distance < 6 cm *n* (%)	13 (16)	15 (18.8)	0.071
Mallampati Score ≥ 3 or mouth opening < 3 cm or thyromental distance < 6 cm *n* (%)	21 (25.9)	24 (30)	0.091
History of Head Neck cancer surgery *n* (%)	11 (13.6)	17 (21.3)	0.203 * 0.068 0.355 *
Cervical radiation *n* (%)	15 (18.5)	17 (21.3)	
Tumor localization *n* (%)			
Oral Cavity	20 (24.7)	22 (27.5)	
Oropharynx	22 (27.2)	27 (33.8)	
Hypopharynx/Larynx	31 (38.3)	21 (26.3)	
Nasopharynx	2 (2.5)	6 (7.5)	
None	6 (7.4)	4 (5.0)	
Cardiovascular disease *n* (%)	42 (51.9)	39 (48.8)	0.062
Hypertension	39 (48.1)	34 (42.5)	
Atrial fibrillation	6 (7.4)	5 (6.3)	
Coronary disease	9 (11.1)	9 (11.3)	
Heart Failure	4 (4.9)	3 (3.8)	

HFJV: high frequency jet ventilation, MVOI: mechanical ventilation with orotracheal intubation, BMI: body mass index (kg/m^2^), ENT: Ear Nose Throat, COPD: chronic obstructive pulmonary disease. SMD: standardized mean difference, SD: standard deviation, * SMD > 0.1: Adjusted variables for the multivariate Analysis.

**Table 2:** Anesthesia Drugs.

	Group 1 (HFJV) *n* = 81	Group 2 (MVOI) *n* = 80	SMD
Neuromuscular Blocker *n* (%)	4 (4.9)	66 (82.5)	2.508
Rocuronium	3 (3.7)	33 (41.3)	
Succinylcholine	1 (1.2)	33 (41.3)	
Dose (mg/kg) (mean, ± SD)	0.63 (±0.26)	0.82 (±0.31)	2.020
Propofol (mean, ± SD)			
Total Dose (mg)	572.8 (±298.73)	410.3 (±258.80)	0.584
Dose (mg/kg/h)	9.29 (±2.92)	6.31 (±3.93)	0.862
Remifentanil (mean, ± SD)			
Total Dose (µg)	259.9 (±146.76)	260.9 (±349.18)	0.294
Dose (µg/kg/h)	4.22 (±1.51)	3.64 (±3.81)	0.685

SMD: standardized mean difference, SD: standard deviation. HFJV: high frequency jet ventilation, MVOI: mechanical ventilation with orotracheal intubation.

## Data Availability

Available on demand.
